# Mechanistic Approaches to Improve Correction of the Most Common Disease-Causing Mutation in Cystic Fibrosis

**DOI:** 10.1371/journal.pone.0155882

**Published:** 2016-05-23

**Authors:** Vedrana Bali, Ahmed Lazrak, Purushotham Guroji, Sadis Matalon, Zsuzsanna Bebok

**Affiliations:** 1 Department of Cell, Developmental and Integrative Biology, University of Alabama at Birmingham, Birmingham, Alabama, United States of America; 2 Department of Anesthesiology and Perioperative Medicine, University of Alabama at Birmingham, Birmingham, Alabama, United States of America; 3 The Cystic Fibrosis Research Center, University of Alabama at Birmingham, Birmingham, Alabama, United States of America; 4 The Lung Injury and Repair Research Center, University of Alabama at Birmingham, Birmingham, Alabama, United States of America; Duke University, UNITED STATES

## Abstract

The most common mutation in the cystic fibrosis transmembrane conductance regulator *(CFTR)* gene leads to deletion of the phenylalanine at position 508 (ΔF508) in the CFTR protein and causes multiple folding and functional defects. Contrary to large-scale efforts by industry and academia, no significant therapeutic benefit has been achieved with a single “corrector”. Therefore, investigations concentrate on drug combinations. Orkambi (Vertex Pharmaceuticals), the first FDA-approved drug for treatment of cystic fibrosis (CF) caused by this mutation, is a combination of a corrector (VX-809) that facilitates ΔF508 CFTR biogenesis and a potentiator (VX-770), which improves its function. Yet, clinical trials utilizing this combination showed only modest therapeutic benefit. The low efficacy Orkambi has been attributed to VX-770-mediated destabilization of VX-809-rescued ΔF508 CFTR. Here we report that the negative effects of VX-770 can be reversed by increasing the half-life of the endoplasmic reticulum (ER) form (band B) of ΔF508 CFTR with another corrector (Corr-4a.) Although Corr-4a alone has only minimal effects on ΔF508 CFTR rescue, it increases the half-life of ΔF508 CFTR band B when it is present during half-life measurements. Our data shows that stabilization of band B ΔF508 CFTR with Corr-4a and simultaneous rescue with VX-809, leads to a >2-fold increase in cAMP-activated, CFTR_inh_-172-inhibited currents compared to VX-809 alone, or VX-809+VX-770. The negative effects of VX-770 and the Corr-4a protection are specific to the native I507-ATT ΔF508 CFTR without affecting the inherently more stable, synonymous variant I507-ATC ΔF508 CFTR. Our studies emphasize that stabilization of ΔF508 CFTR band B in the ER might improve its functional rescue by Orkambi.

## Introduction

**Fig 1:**
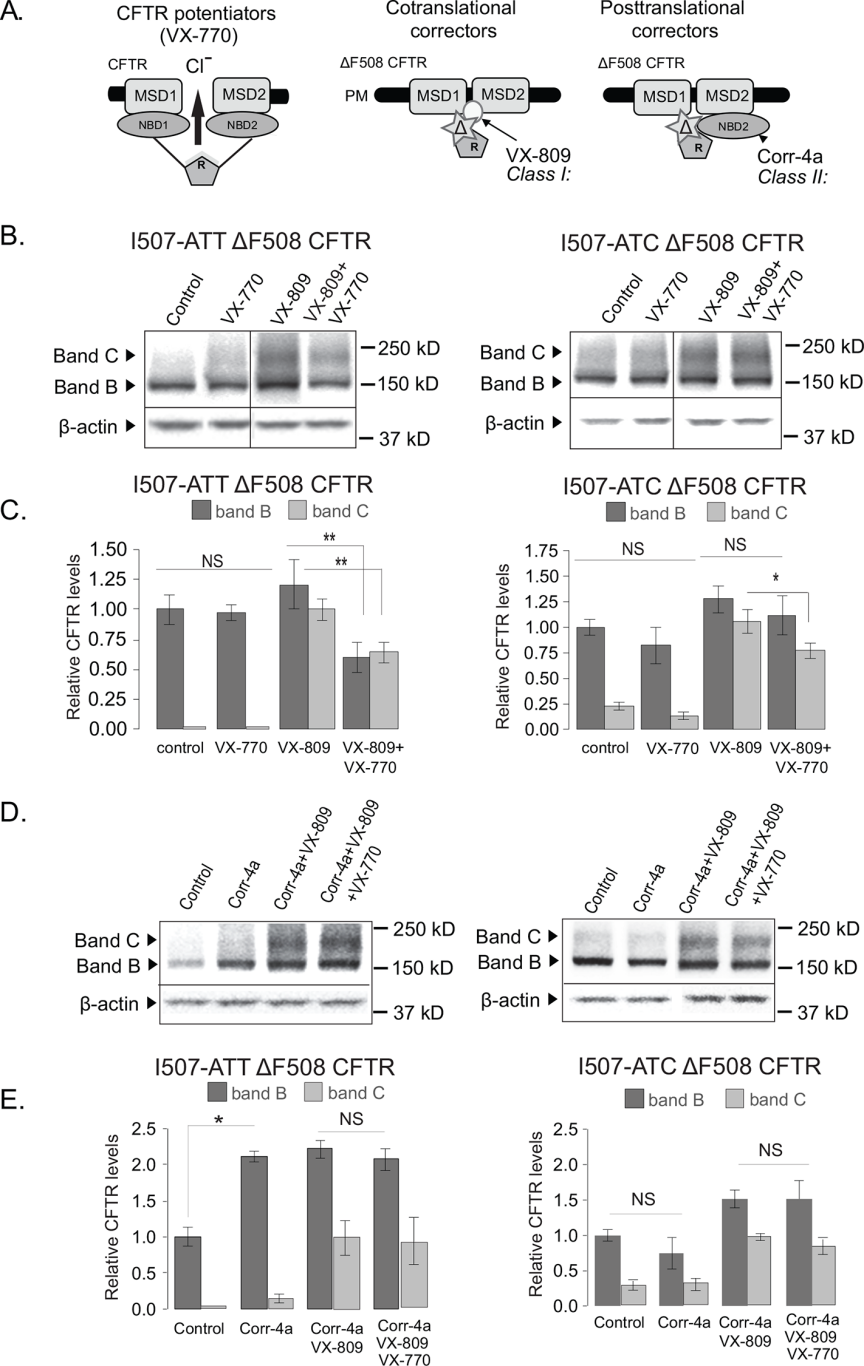
Corr-4a diminishes negative effects of VX-770 on VX-809-rescued I507-ATT ΔF508 CFTR. **A.** Mechanism of action of CFTR modulators used in our studies. **B.** Western blot analysis of I507-ATT and I507-ATC ΔF508 CFTR expressing HEK-293 cell lysates following vehicle control (0.15% DMSO, 16 h, 37°C) or corrector treatment (5μM VX-809, 5μM VX-770 and 5μM VX-809+5μM VX-770, 16 h, 37°C). Loading control: β-actin. **C.** Densitometry of band B and band C I507-ATT and I507-ATC ΔF508 CFTR levels. CFTR band B was plotted relative to vehicle control (DMSO). Band C was plotted relative to VX-809 (37°C). DMSO control, corrector VX-809 (VX-809), potentiator VX-770 (VX-770), combination (VX-809+VX-770), n = 9,9,6,3. **D.** Western blot analysis of I507-ATT and I507-ATC ΔF508 CFTR expressing HEK-293 cell lysates following vehicle control (0.2% DMSO, 24 h, 37°C), Corr-4a (10μM, 24 h, 37°C) or drug combination treatments (5μM VX-809+10 μM Corr-4a and 5μM VX-809+10 μM Corr-4a+5μM VX-770, 16 h, 37°C). Loading control: β-actin. **E.** Quantification of I507-ATT and I507-ATC ΔF508 CFTR levels. CFTR band B levels were plotted relative to vehicle control (DMSO). Band C levels were plotted relative to VX-809 (37°C). DMSO control (Ctr), corrector combination (VX-809+Corr-4a), combination of correctors and potentiator VX-770 (VX-809+Corr-4a+VX-770), n = 4. All values are means ± 1 SD. *p<0.05; **: p<0.02, significantly different from VX-809-treated sample by ANOVA, Turkey-Kramer procedure.

The most common cause of cystic fibrosis (CF) is the out-of-frame deletion of three nucleotides (CTT) in the *CFTR* gene, resulting in loss of phenylalanine at position 508 (ΔF508) of the CFTR protein and a synonymous mutation (ATC/ATT) at codon encoding isoleucine 507 [1–3]. The mutant protein is misfolded and subjected to endoplasmic reticulum associated degradation (ERAD) [4]. When rescued from ERAD, ΔF508 CFTR demonstrates reduced plasma membrane stability and functional abnormalities [5]. Efforts to treat CF caused by the ΔF508 mutation focus on finding small molecular correctors that enhance ΔF508 CFTR folding co- and/or post-translationally and potentiators to improve its function (Fig 1A) [5, 6]. Orkambi (Vertex Pharmaceuticals), the first FDA approved combinational treatment for CF contains the cyclopropane carboxamide derivative corrector (VX-809, Lumacaftor) and the *N*-(2,4-Di-*tert*-butyl-5-hydroxyphenyl)-4-oxo-1,4-dihydroquinoline-3-carboxamide potentiator (VX-770, Ivacaftor). It is estimated that 50% of CF patients will benefit from combination therapy [7].

Orkambi has been developed based on the results of a phase 2 randomized controlled clinical trial indicating that treatment with VX-809 and VX-770 improved lung function as measured by FEV1 in CF patients, homozygous for the ΔF508 mutation, with a modest effect on sweat chloride concentration [8]. Yet, two independent groups reported that chronic co-administration of these compounds destabilized the low temperature (27°C) and VX-809-corrected ΔF508 CFTR [9, 10]. Such findings explain the lower than expected therapeutic effect of Orkambi, observed in multiple clinical trials [11]. Consequently, high throughput screening identified new potentiators that do not interfere with correctors [12]. Moreover, dual activity compounds that act as both correctors and potentiators, such as aminoarylthiazoles, represent another advancement in treatment [13].

Studies utilizing second-site suppressor mutations indicate that at least two of the folding defects need to be corrected simultaneously to achieve significant rescue of ΔF508 CFTR and suggest that corrector combinations may be used for that purpose [14]. Therefore, additive and synergistic effects of corrector combinations have been studied extensively [15–19]. Although the number of CFTR modulators is rapidly growing and reports indicate that the binding of these is CFTR-specific, it is not clear whether they distinguish between certain genetic variants or folding intermediates. Because drug combinations have therapeutic potential, studies analyzing the specificity and mechanism of action of CFTR modulators are necessary to develop efficient therapeutic combinations [20]. In addition to corrector and potentiator combinations that target CFTR folding and function, Roberts *et al*., proposed an orthogonal method to stabilize ΔF508 CFTR. They designed peptides to inhibit the binding of the rescued ΔF508 CFTR to a component of the cell surface protein quality control machinery, CAL [21]. They suggest that combining ΔF508 CFTR stabilizers with correctors and potentiators could provide novel therapeutic cocktails with greater therapeutic benefit.

We have demonstrated that the I507-ATC/ATT synonymous codon change contributes to the misfolding [22] and functional defects of ΔF508 CFTR [23]. In a follow up study, we determined that while VX-809 corrected both variants, the beneficial effects of the bisaminomethylbithiazole compound Corr-4a were specific to the native, I507-ATT ΔF508 [24]. We have also demonstrated that Corr-4a+VX809 combination enhanced rescue efficiency [24]. Considering these results, we hypothesized that stabilization of ΔF508 CFTR band B with Corr-4a counteracts the negative effects of VX-770 on VX-809-rescued ΔF508 CFTR. Using HEK-293 cells stably expressing ΔF508 CFTR, we observed similar negative effects of VX-770 on VX-809-rescued native ΔF508 CFTR as others reported in either heterologous cell lines or primary cells [9, 10]. We used the I507-ATC ΔF508 CFTR variant that contains the wild type CFTR codon at the I507 location, as control. Here we report that addition of Corr-4a to the VX-809+VX-770 combination reverses the negative effects of VX-770 and results in a >2-fold increase in cAMP-activated CFTR_inh_-172- inhibited whole cell currents across HEK-cells stably expressing the native, I507-ATT ΔF508 CFTR but not its synonymous variant.

## Materials and Methods

### Cell lines

Single cell clones of human embryonic kidney 293 (HEK-293) cells stably expressing WT CFTR, I507-ATT or I507-ATC ΔF508 CFTR were developed and maintained as described [22–24].

### Treatment with CFTR potentiator and ΔF508 CFTR corrector compounds

VX-770 and corrector VX-809 (Selleck Chemicals, Houston, TX, USA) were used at 5 μM for 16 hours at 37°C. Corrector Corr-4a (CFTR Compound Program (Rosalind Franklin University of Medicine and Science, Chicago, IL, USA) and CFFT (Bethesda, MD, USA)) was used at 10 μM for 16 hours. VX-809 and Corr-4a were present during the experiments, but VX-770 was washed out during patch clamp recordings. Tissue culture grade dimethyl sulfoxide (DMSO, Sigma Aldrich, St. Louis, MO, USA) was added as control for 16 hours at 37°C. at 0.15–0.2%, corresponding to the concentration of DMSO in the corrector stocks.

### CFTR expression

Western blots were performed as previously described [22–24].

### CFTR turnover

CFTR stability was determined following cycloheximide block (0.2 mg/ml) using following formula: half-life equals elapsed time multiplied by logarithm of two, divided by logarithm of ratio of beginning amount and ending amount (t_1/2_ = (elapsed time * log2) / log (beginning amount/ending amount)), as described [23, 24]. Cells were pretreated with CFTR correctors/potentiators for 16 hours and the compounds were present during the chase period.

### Whole-cell patch clamp

Experiments were performed at room temperature as described previously [24], with the following modifications: cells were treated with correctors (VX-809 and Corr-4a) and a potentiator (VX-770) as specified above, but VX-770 was removed during patch-clamp recordings. Data are presented as forskolin+IBMX-activated and CFTR_inh_-172 (20 μM), inhibited maximum currents, where each recording was divided by the cell capacitance (ΔpA/pF). Results were plotted as mean±SE using data analysis and graphing software IgorPro 6.37 (WaveMetrics, Lake Oswego, OR USA).

## Results

### Combination treatment with VX-809+VX-770 reduces I507-ATT ΔF508 CFTR levels compared to VX-809 alone, at physiological temperature

Previous studies have shown that chronic (16 h) treatment of multiple ΔF508 CFTR expressing cell types with VX-770+VX-809 combination diminished the corrective effects of VX-809 by increasing the turnover and reducing the plasma membrane levels of rescued ΔF508 CFTR [9, 10]. In those studies, pharmacological rescue of ΔF508 CFTR was aided by low temperature (27°C) culture. To circumvent the undefined consequences of culturing cells at low temperature [25], we analyzed the effects of VX-809 and VX-770 individually and in combination at physiological temperature (37°C), using the same dosage of the corrector and potentiator as previously reported [9, 10] (Fig 1).

We demonstrate that VX-770 alone (5μM for 16h) does not alter steady state I507-ATT, or I507-ATC ΔF508 CFTR levels (Fig 1B and 1C). Consistent with previous results [24], VX-809 treatment (5μM for 16h) resulted in significant rescue of fully processed band C CFTR for both variants. When cells were treated with VX-770 and VX-809 combination (5μM each for 16h) we observed a considerable reduction (40%) in both band B and C levels of native, I507-ATT ΔF508 CFTR (Fig 1B and 1C, left). In contrast, no changes were found in I507-ATC ΔF508 CFTR band B levels and only 20% band C reduction was seen (Fig 1B and 1C, right). These results suggest that VX-770 destabilizes the native ΔF508 CFTR.

### Stabilization of I507-ATT ΔF508 CFTR by Corr-4a offsets the negative effects of VX-770

We have reported that in the presence of Corr-4a the half-life of I507-ATT ΔF508 CFTR band B increased from <30 min to 100 min, similar to the half-life of the I507-ATC ΔF508 CFTR under control conditions. Corr-4a had to be present during the life span of ΔF508 CFTR to elicit the stabilizing effect. Further, Corr-4a+VX-809 combination significantly increased rescue efficiency compared to Corr-4a or VX-809 alone [24]. Based on these results, we hypothesized that stabilization of ΔF508 CFTR band B with Corr-4a could reverse the negative effects of VX-770 on the VX-809-rescued ΔF508 CFTR. Furthermore, while additive effects of the VX-809+Corr-4a combination was documented in HEK-293 [24]. and other epithelial cell lines expressing ΔF508 CFTR [17], as well as in samples from CF patients homozygous for ΔF508 CFTR [16, 18], the Corr-4a+VX-809+VX-770 treatment combination has not been investigated.

Therefore, in the next set of experiments, we concentrated on how stabilization of band B with Corr-4a influences the effects of VX-809+VX-770. Consistent with our previous findings [24], Corr-4a (10μM, 16 h at 37°C) increases I507-ATT ΔF508 CFTR band B levels (2-fold) without affecting band C levels (Fig 1D, left, lane 2 and E). As expected, treatment with VX-809+Corr-4a resulted in significantly higher band B and C levels (Fig 1D, left, lane 3). More importantly, in contrast to VX-809+VX-770 treatment (Fig 1B, lanes 3–4 and C, left), addition of VX-770 to the VX-809+Corr-4a combination did not reduce I507-ATT ΔF508 CFTR band B or Band C (Fig 1D, lanes 3–4 and E, left). As previously [24], we did not observe significant changes in I507-ATC ΔF508 CFTR band B levels following Corr-4a treatment. Furthermore, there were no significant differences in band B or band C levels following Corr-4a+VX-809 or Corr-4a+VX-89+VX-770 treatment in I507-ATC ΔF508 CFTR expressing cells (Fig 1D and 1E, right).

### VX-770 reduces the half-life of VX-809-recued ΔF508 CFTR

**Fig 2:**
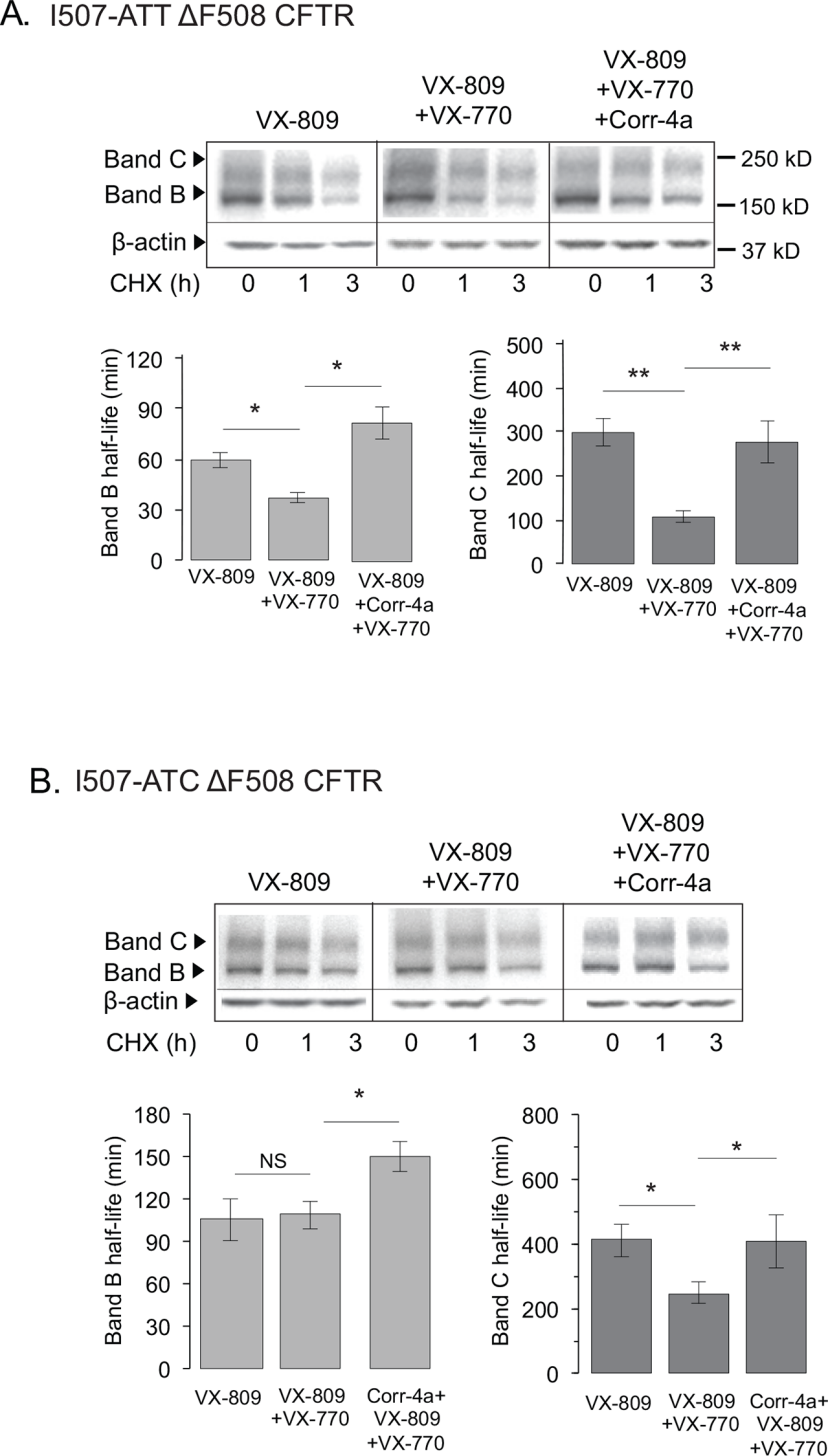
Corr-4a reverses the negative effects of VX-770 on VX-809-corrected ΔF508 CFTR half-life. **A.** I507-ATT ΔF508 CFTR turnover in VX-809 (5μM), VX-809+VX-770 (5μM+5μM) and VX-809+VX-770+Corr-4a (5μM +5μM+10μM) treated cells. **B.** I507-ATC ΔF508 CFTR turnover in VX-809 (5μM), VX-809+VX-770 (5μM+5μM) and VX-809+VX-770+Corr-4a (5μM +5μM+10μM) treated cells. Cycloheximide (CHX) (200μg/ml, 37°C) was used to inhibit protein synthesis. Cells were lysed at 0, 1 and 3 hours post CHX treatment. Representative gels are shown on top. Loading control: β-actin. CFTR band B (left) and band C (right) half-lives are plotted in the lower panels. Please see text for half-life calculation explanation. All values are means ± 1 SD. *p<0.05; **: p<0.02, significantly different by ANOVA, n = 3.

To understand the mechanisms by which Corr-4a counteracts the negative effects of the VX-770 potentiator on VX-809-rescued ΔF508 CFTR, we analyzed the turnover of the I507-ATT and I507-ATC ΔF508 CFTR band B and C, using cycloheximide protein synthesis block (CHX, (200μg/ml for 1h to 3h) [24]. The compounds were present during the course of the experiments at the same concentrations as during the pre-treatment. These conditions were chosen based on our previous observations that the effects of Corr-4a and VX-809 diminished shortly if they were not present during the chase period. This suggests that while the compounds primarily correct the ER form, their presence is necessary for stabilization during the life span of the protein [23, 24] (Fig 2). In the presence of VX-809 alone, I507-ATT ΔF508 CFTR band B half-life was 60 min ± 5min, n = 3). Addition of VX-770 to VX-809 reduced the half-life to 30 min± 3 min, n = 3). However, in Corr-4a+VX-809+VX-770 treated cells the half-life of I507-ATT ΔF508 CFTR band B was higher (80 min ± 10min, n = 3) than following VX-809 treatment alone. We observed a similar negative effect on band C stability when the cells were treated with VX-809+VX-770, but even a larger (300 min to 100 min, ± 32min and 12 min, respectively, n = 3) reduction in band C half-life. Importantly, addition of Corr-4a (5μM, 16h at 37°C) reversed the negative effects of VX-770 on I507-ATT ΔF508 CFTR band C half-lives as well (277min ± 47min, n = 3). VX-809+VX-770 combination did not reduce the half-life of the I507-ATC ΔF508 CFTR band B (105min ± 15min, n = 3), tested as control. Addition of corr-4a conferred further stabilization as demonstrated by increased band C half-life (150min ± 11min, n = 3). These results imply that the destabilizing effects of VX-770 are determined by conformational changes caused by the I507-ATC>ATT synonymous codon change rather than the amino acid sequence. Specifically, the negative effects of VX-770 are specific to the native ΔF508 CFTR and do not affect the variant ΔF508 CFTR in which I507 is encoded by the wild type codon (see discussion).

### Corr-4a in combination with VX-809+VX-770 increases cAMP-activated and CFTR_inh_-172-inhibitable currents in native (I507-ATT) ΔF508 CFTR expressing cells

**Fig 3:**
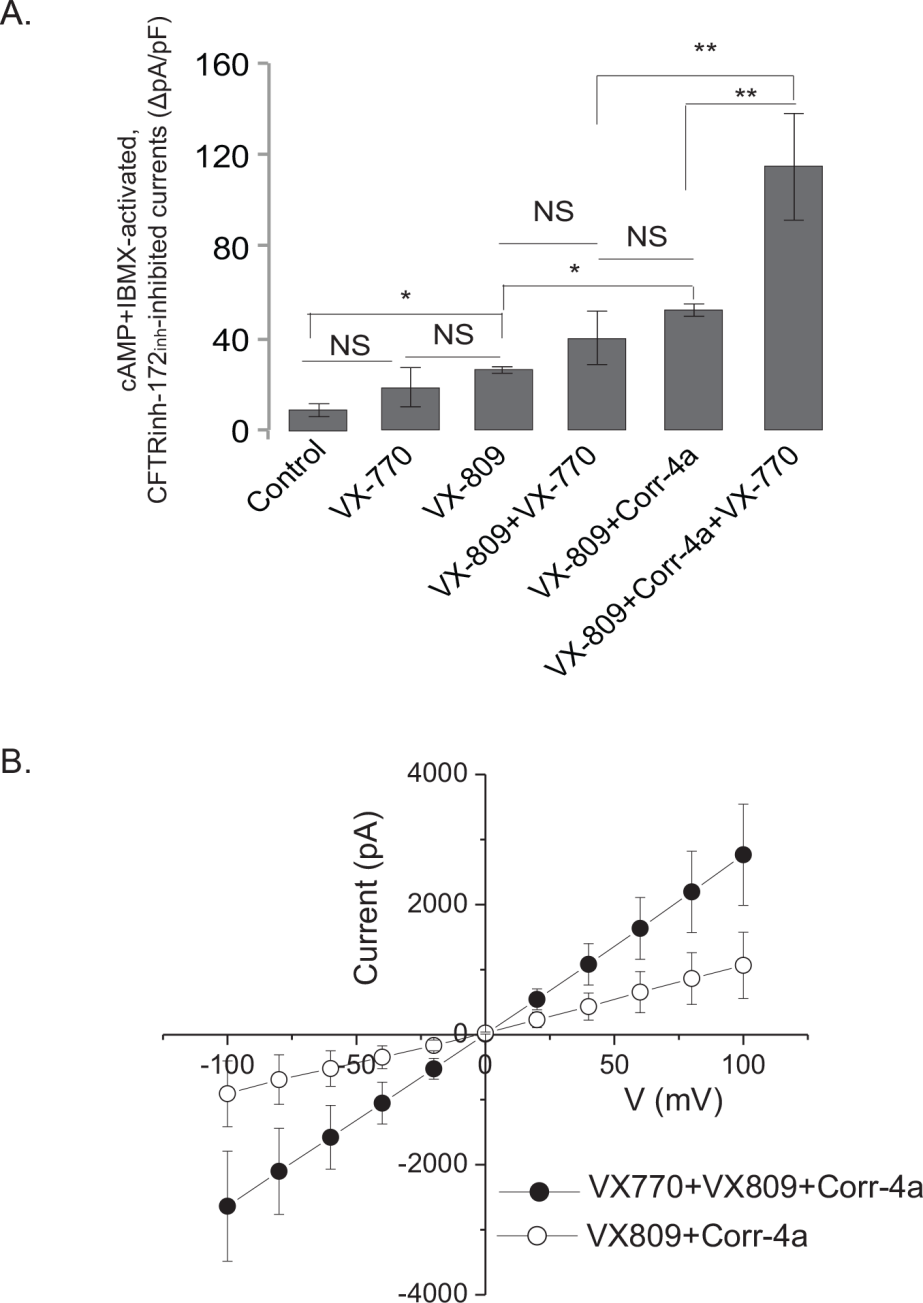
Corr-4a increases cAMP-activated, CFTR_inh_-172-inhibited whole-cell currents in VX-809+VX-770 treated I507-ATT ΔF508 CFTR expressing cells. Whole-cell patch clamp studies were performed following treatment with CFTR modulators as specified (5μM VX-809 + 10μM Corr-4a+ 5μM VX-770, 16h, 37°C). Corr-4a and VX-809 were present during recordings, but VX-770 was washed out prior to experiments. **A:** Results are plotted as maximum forskolin+IBMX-induced and CFTR_inh_-172-inhibited maximum currents (ΔpA/pF), n = 21 (Control, VX-809 and VX-809+Corr-4a), n = 13 (VX-770, VX-809+VX-770 and VX-809+VX-770+Corr-4a), *: p<0.01; **: p<0.001 by ANOVA; error bars: means± SE **B:** Representative I/V relationships obtained from cells treated with VX-809+Corr-4a and VX-809+Corr-4a+VX-770, n = 8, error bars: means± SE.

We performed whole-cell patch clamp studies to determine the functional consequences of chronic (16 h) VX-770 co-treatment during I507-ATT ΔF508 CFTR rescue with VX-809 and Corr-4a. After recording baseline currents, we perfused cells with the bath solution containing forskolin (10 μM) and IBMX (100 μM) until currents reached plateau values. Then cells were perfused with a bath solution containing forskolin, IBMX and the CFTR inhibitor CFTR_inh_-172 (20 μM), to inhibit currents. We calculated maximum forskolin+IBMX-induced currents that were inhibited with CFTR_inh_-172. Notably, we washed VX-770 out prior to patch-clamp recordings because the presence of VX-770 during recordings caused a significant variability in cAMP+IBMX-activated whole-cell currents (data not shown). When cells were pretreated with VX-770, but the compound was removed during the recordings, we did not see significant functional effects compared to untreated controls (*Fig 3).* When cells were pretreated with VX-809 alone, we measured similar cAMP+IBMX-induced ΔF508 CFTR currents as in previous studies (31.71±3.43 pA/pF, n = 21) [24]. Interestingly, and contrary to the biochemical data demonstrating 40% reduction in CFTR levels (Fig 1), cAMP+IBMX-induced whole-cell currents did not change significantly when cells were pretreated with VX-809+VX-770 combination (Fig 3A). In agreement with our previous results [24], when cells were treated with VX-809+Corr-4a, we recorded significantly higher (53.0±5.4 pA/pF, n = 21) cAMP+IBMX-activated currents than following VX-809 alone (Fig 3A). We did not test Corr-4a alone because treatment with this corrector did not result in significant functional rescue of ΔF508 CFTR [24]. Most importantly, following VX-809+Corr-4a+VX-770 pretreatment, maximum cAMP-activated ΔF508 CFTR currents were 2-fold higher (112.0±22.3 pA/pF, n = 13) than in the presence of VX-809 or VX-809+VX-770 (Fig 3A and 3B). Notably, the functional increase was more significant than it would be expected from the protein levels. These results are consistent with the idea that stabilization of band B ΔF508 CFTR with class II correctors such as Corr-4a postranslationally, corrects functional defects in addition to improving protein stability.

## Discussion

Here we demonstrate that the previously observed negative effects of chronic VX-770 co-administration on VX-809-rescued ΔF508 CFTR [9, 10] can be reversed by enhancing the half-life of the mutant band B and stabilizing the protein continuously throughout its life span with Corr-4a co-treatment. Furthermore, Corr-4a co-treatment significantly enhances the functionality of the rescued ΔF508 CFTR when it is co-administered with VX-809 and VX-770. We chose Corr-4a co-treatment with VX-809+VX-770 for these studies based on our previous finding that it specifically stabilizes the native, I507-ATT ΔF508 CFTR band B when it is present continuously in the cells. However, the stabilizing effect of Corr-4a rapidly diminished when it was removed from the cells [24]. This may be the reason why one group reported no significant effects of Corr-4a on ΔF508 CFTR [15].

Additional studies have demonstrated that the effects of Corr-4a are not CFTR specific since it corrects folding mutants of hERG and P-gp as well [18]. Interestingly, our studies indicate that Corr-4a distinguishes between the native (I507-ATT), and the I507-ATC ΔF508 CFTR synonymous variants when it is administered alone, yet simultaneous treatment with VX-809 resulted in additive effects on both variants [24]. This suggests that correction with VX-809 enhances the levels of folding intermediates that are stabilized by Corr-4a. Moreover, additive effects of Corr-4a and VX-809 combination were also reported in ΔF508 CFTR homozygous human primary bronchial epithelial cells [18], as well as in CFBE41o- cells expressing ΔF508 CFTR and organoid samples derived from CF patients homozygous for ΔF508 mutation [16], supporting the idea that Corr-4 has corrector capability. Nevertheless, these studies also suggest that while the primary mechanism of VX-809 is cotranslational and Corr-4a is posttranslational [26], it is most likely that they act on multiple folding intermediates.

Although numerous ΔF508 CFTR corrector and potentiator combinations have been analyzed [6, 10, 19, 26–29], according to our best knowledge, the effects of this particular combination (VX-809+Corr-4a+VX-770) have not been analyzed in a mechanistic fashion. In addition to recapitulating the results of previous reports indicating reduced total ΔF508 CFTR protein levels following VX-809 and VX-770 co-treatment in airway cells [23, 24], we demonstrate that VX-770 increases the turnover of both the core glycosylated (band B) and fully processed (band C) of the native, I507-ATT ΔF508 CFTR. Because VX-770 alone had no considerable effect on ΔF508 CFTR levels, this implies that VX-770 targets the VX-809 “corrected” ΔF508 CFTR soon after completion of translation, in the early secretory pathway, since the primary mechanism of VX-809 action is cotranslational [16, 28]. Indeed, increasing the stability of band B with posttranslationally acting Corr-4a was sufficient to diminish the negative effects of VX-770 on protein levels.

In contrast to Ussing chamber studies by Cholon *et al* [9], in which they observed significant reduction in CFTR function following co-administration of VX-809+VX-770 compared to VX-809 alone, no changes in rescued ΔF508 CFTR function were seen at the single cell level. This may be due to different experimental conditions. Specifically, we did not incubate cells at low temperature to aid ΔF508 CFTR rescue. Importantly, low temperature-rescued ΔF508 CFTR loses its function rapidly [25] and when combination of low temperature and VX-809 were tested [9, 10], the functional reduction following VX-770+VX-809 co-treatment might have resulted from the functional instability of low temperature rescued ΔF508 CFTR. Having said that, we can’t exclude that the differences in cellular background may also contribute to the differences in functional results observed following chronic VX-770+VX-809 treatment by us, and others [9, 10]. Furthermore, it is also possible that a 40% reduction in total CFTR expression following VX-809 and VX-770 co-treatment may not be sufficient to impair ΔF508 CFTR function at the cellular level (patch clamp) when traces of the potentiator (VX-770) are likely to be present in the cells. Additionally, according to our previous experience, CFTR protein levels have to decrease significantly (>50%) to measure significant functional differences [30].

However, it is more important to consider the therapeutic potential of band B ΔF508 CFTR stabilization that we observed following co-administration of Corr-4a with VX-809 and VX-770 (>2-fold increase in cAMP-activated Cl^-^ currents) when cells were treated with the CFTR modulators at physiological temperature (37°C). These results demonstrate for the first time that stabilization of ΔF508 CFTR with Corr-4a enhances the functionality of the VX-809-rescued ΔF508 CFTR when VX-770 is present during rescue.

Compared to native ΔF508 CFTR, the negative effects of VX-770 were minor on the I507-ATC ΔF508 CFTR variant tested as control. These results are consistent with the idea that the higher stability [24] and alternative folding state [23] of the I507-ATC ΔF508 CFTR render it resistant to VX-770-mediated destabilization. Indeed, we have shown that Corr-4a confers similar level of stabilization to ΔF508 CFTR as the I507-ATC (wild type) codon [24]. Taking into account previously reported conformational differences between I507-ATT and I507-ATC ΔF508 CFTR [23] and their distinct responses to ΔF508 correctors [24], the results presented here demonstrate that VX-770 elicits it’s primary negative effect on folding intermediates that can be corrected by Corr-4a or possibly by other correctors that stabilize the ER, band B form of ΔF508 CFTR.

Considering that the stabilizing effects of Corr-4a and the synonymous codon change (I507-ATT>ATC) in ΔF508 CFTR are similar, one possibility is that they alter the recognition of the rescued protein by the cell surface quality control machinery. For example, it has been demonstrated that the Golgi-associated PDZ domain protein CAL directs rescued ΔF508 CFTR to early lysosomal degradation by binding to the PDZ-interacting sequences at the C-terminus of CFTR [31–35]. Inhibition of this binding with CAL-binding inhibitor peptides such as kCAL01 stabilizes ΔF508 CFTR [21]. However, if a corrector combination increases the cell surface stability of ΔF508 CFTR by reducing its binding affinity to CAL, it is possible that the effects of stabilizing peptides that competitively inhibit CAL-mediated degradation of ΔF508 CFTR, such as kCAL1, may be reduced as well. In contrast, if the ΔF508 CFTR stabilizing effects of peptides remain significant, such biologics may further improve the efficiency of correctors. Nevertheless, combinatorial stability tests with CAL PDZ-domain inhibitors, ΔF508 CFTR correctors, and potentially other stabilizing molecules could identify novel cocktails with greater therapeutic benefit.

In agreement with other reports highlighting the context-dependent effects of CFTR modulators [10], our studies reinforce that the I507 codon plays important role in ΔF508 CFTR folding and responsiveness to pharmacological modulators [22, 23]. Indeed, similar to some second site mutations [10] the I507-ATC/ATT mutation renders native ΔF508 CFTR sensitive to further destabilization by VX-770. Having said that, Cholon *et al*. proposed that while VX-770-dependent destabilization of CFTR is favorable in the context of the G551D mutation, its original target, destabilization of the already unstable I507-ATT ΔF508 CFTR has negative effects on its biogenesis and function [9]. Interestingly, the I507-ATC ΔF508 CFTR, used as control in our studies, does not share this phenotype.

Taken together, our results are consistent with the hypothesis that stabilization of the ER, band B form of ΔF508 CFTR is necessary to obtain sufficient biochemical and more importantly, functional rescue. Furthermore, we provide additional evidence that the I507-ATC/ATT codon change contributes to the biochemical instability of ΔF508 CFTR [22–24]. Considering that synonymous codon usage [36] and factors modulating intrinsic folding propensity can also alter CFTR expression levels [37] it is clear that synonymous mutations can also influence protein structure and function (for review: [38]). We highlight an additional layer of complexity accompanying the ΔF508 CFTR mutation and bring attention to the potential significance of ΔF508 CFTR correctors that stabilize band B CFTR as possible components of combination therapeutics for CF.
